# Paraneoplastic Leukocytosis and Thrombocytosis as Prognostic Biomarkers in Non-small Cell Lung Cancer

**DOI:** 10.3779/j.issn.1009-3419.2016.11.02

**Published:** 2016-11-20

**Authors:** Prajwal BODDU, Dana VILLLINES, Mebea AKLILU

**Affiliations:** 1 Hematology/Oncology, Advocate Creticos Cancer Center, Chicago, IL 60657, USA; 2 Department of Clinical Research, Advocate Illinois Masonic Hospital, Chicago, IL 60657, USA; 3 Department of Internal Medicine, Advocate Illinois Masonic Medical Center, Chicago, IL 60657, USA

**Keywords:** Paraneoplastic, Leukothrombocytosis, Paraneoplastic leukocytosis, Paraneoplastic thrombocytosis, Lung neoplasms, Prognosis, Cytokine, Epidermal growth factor receptor

## Abstract

**Background and Objectives:**

Search for inexpensive laboratory markers have identified associations between blood counts and lung cancer outcomes. In this study, we evaluated the prognostic value of paraneoplastic leukocytosis (p-Leukocytosis) and paraneoplastic thrombocytosis (p-Thrombocytosis) in patients with non-small cell lung cancer (NSCLC). We also studied their relation to the expression of commonly detected molecular markers.

**Methods:**

We conducted a retrospective chart review on 571 consecutive NSCLC patients over a 10 year period. Blood counts were recorded at the time of cancer diagnosis. *Kaplan-Meier* survival curves were used to compare overall survival (OS) between patients with and without p-Leukocytosis (or) p-Thrombocytosis (p-Leuko/Thrombocytosis). *Cox* regression was used to determine if leukocytosis/thrombocytosis was a predictor of OS in NSCLC.

**Results:**

Patients with p-Leukocytosis and p-Thrombocytosis had a significantly poorer survival compared patients with normal blood counts (*P* < 0.001). In a multivariate survival analysis, both continued to correlate even when adjusted for histology, gender, stage and chemotherapy (*P* < 0.01, 0.03 respectively). Stage Ⅰ and Ⅱ NSCLC with p-Leuko/Thrombocytosis did not perform poorly compared to stage Ⅰ/Ⅱ NSCLC patients without paraneoplasia. Patients with the combined leukothrombocytosis syndrome did not have worse outcomes compared to those with either paraneoplastic syndrome alone.

**Conclusions:**

p-Leuko/Thrombocytosis is an accessible laboratory parameter of prognostic value in NSCLC. Evidence of p-Leuko/Thrombocytosis portends poor survival. The role of various cytokines in tumor pathobiology provides a rationale for identifying cytokine factors responsible for the paraneoplasia and administering anti-cytokine therapies alongside traditional chemotherapy in an attempt to improve survival outcomes in these subset of patients.

## Introduction

Non-small cell lung cancer (NSCLC) constitutes about 85% of cases of lung cancer, a leading cause of cancer with a dismal 5 year survival rate of only 22%^[1]^. There are numerous tumor and host related prognostic markers identified in NSCLC among which stage, performance status and gender are best defined^[2]^. There is ongoing search for inexpensive, easily available prognostic and predictive biomarkers.

Cancer associated hematopoiesis may affect one or all cell lines and, by various postulated mechanisms, may influence tumor progression and suppression of tumor immunity^[3]^. Leukocytosis and thrombocytosis have been associated with inferior prognosis^[4-7]^. Tumor associated Leukocytosis, is not only an early poor prognostic feature, but also helps differentiate malignant and benign lesions^[5]^. NSCLC may secrete one or more cytokine factors resulting in varied hematological paraneoplastic syndromes. Granulocyte-macrophage colony-stimulating factor (GM-CSF), granulocyte-CSF (G-CSF), parathyroid hormone-related peptide, interleukin (IL)-1, IL-6, and tumor necrosis factor (TNF) production have been implicated to be responsible for these hematological paraneoplastic syndromes^[4-9]^. Isolated cases of paraneoplastic eosinophilia in lung cancer have been reported and have been associated with very poor outcomes^[8]^. White blood cells (WBCs) and platelets are among the frequently ordered blood cell counts and may serve as useful prognostic markers at the time of diagnosis to prognosticate and tailor therapy decisions. In this study, we intend to evaluate the prognostic value of leukocytosis and thrombocytosis in patients with NSCLC and also study their relation to the commonly detected molecular markers. Furthermore, the association of leukocytosis with thrombocytosis as a combined paraneoplastic syndrome will be studied.

## Materials and methods

This is a retrospective chart review of patients with a diagnosis of NSCLC from January 2005 to December 2015. Data was abstracted from our institution's Cancer Center registry with supplemental chart review as necessary. Institutional Review Board approval was obtained prior to study conduct.

Data was abstracted for 571 consecutive patients treated in Advocate Illinois Masonic Medical Center, our community-based teaching institution. The study variables detailed below were abstracted from our institution's cancer registry database: age, gender, histology type, molecular receptor status (if applicable), tumor grade, tumor necrosis (radiologic or histologic), overall survival (OS), and blood counts. Blood counts were measured at the time the patient's NSCLC was first diagnosed. A WBC count between 4, 000/mm^3^-12, 000/mm^3^ and a platelet count between 150, 000/mm^3^-450, 000/mm^3^ was considered normal ranges for the study. Paraneoplastic thrombocytosis (p-Thrombocytosis or Thrombocytosis) was defined as platelet count than 450, 000/mm^3^ after excluding inflammatory disease, autoimmune disease, any other coexistent malignancy other than lung cancer, post splenectomy states. Paraneoplastic leukocytosis (p-Leukocytosis or Leukocytosis) was defined as WBC counts greater than 12, 000/mm^3^ with no evidence of infection or no improvement in white blood cell count after at least 7 d of empiric antibiotic therapy, steroid use before or concurrent with WBC rise, acute inflammatory process, active connective-tissue disease, post splenectomy states, or rise in WBC counts secondary to non-neutrophil white blood cell lines. P-Leukothrombocytosis was defined as having both p-Leukocytosis and p-Thrombocytosis in the same patient. Blood counts were recorded at the time of cancer diagnosis.

### Analysis plan

Descriptive statistics are presented as means and standard deviations for continuous data and as counts and percentages for dichotomous and categorical data. Survival curves with *Kaplan-Meier* estimates were used to determine compare OS between groups with and without leukocytosis/thrombocytosis. Comparison of survival curves was done using the *Log-rank* test. *Cox* regression was used to determine if leukocytosis-thrombocytosis is a predictor of OS in NSCLC patients. Variables were included in the multivariate analysis if the *P*-value was < 0.10 with the exception of 'age' as the hazard ratio and confidence intervals indicated there was very little real-world predictive value. Analysis will be performed using SPSS®21 (Chicago, IL, 2012) and statistical significance will be determined at *P* < 0.05.

## Results

### Initial patient characteristics

Of 571 patients identified, 11.5% of the patients (66 patients) with p-Thrombocytosis, p-Leukocytosis or both. Baseline patient characteristics are shown in Table 1. The age [(65.4±10.9) *vs* (66.4±10.5)] and gender (male 60.9% *vs* 57%) characteristics of this subgroup were similar compared to the NSCLC patients without Leuko/thrombocytosis (Table 1). Leuko/thrombocytosis was found in 1.3% of stage Ⅰ/Ⅱ and 10.5% of stage Ⅲ/Ⅳ NSCLC patients.

**1:** Baseline characteristics of study population (*n*=571)

Variables	Patients without para L/T (*n*=505)	Patients with para L/T (*n*=66)
Paraneoplastic thrombocytosis (*n*)	-	26
Paraneoplastic leukocytosis (*n*)	-	50
Leukothrombocytosis (*n*)	-	10
Age (mean in years)	66.4 (55.9-66.9)	65.4 (54.5-76.3)
Male (%)	57	60.9
Histology		
Adeno (%)	44.9	27.3
Squamous cell (%)	31.1	33.3
Large cell (%)	3.5	4.5
Bronchoalveolar (%)	1.2	4.5
Adenosquamous (%)	1.5	0
Non-small, no defined histology (%)	17.6	30.3
Stage		
Ⅰ (%)	23.5	6.2
Ⅱ (%)	8.6	4.6
Ⅲ (%)	25.1	17
Ⅳ (%)	42.9	72.3
*EGFR* status (proportion)	4/51	1/9
*ALK* mutation status (proportion)	2/51	0/9
Gross or microscopic necrosis (%)	-	11.8
Chemotherapy-administered (%)	50.2	33
para L/T: paraneoplastic Leuko/thrombocytosis; EGFR: epidermal growth factor receptor.		

### Between group differences

Differences in clinic-pathological and survival variables of groups with and without paraneoplastic Leuko/thrombocytosis (p-Leuko/Thrombocytosis) is shown in Table 2. Patients with p-Leuko/Thrombocytosis did not differ from patients without p-Leuko/Thrombocytosis in age or tumor grade but differed significantly in stage and mean OS (Table 2). Necrosis was observed in only 11.8% of patients with leukocytosis (*P*=0.6).

**2:** Between group differences of patients without and with p-Leuko/thrombocytosis

	Patients without para L/T (*n*=505)	Patients with para L/T (*n*=66)	*P* value
Age (mean in years)	66.3±10.5	64.9±10.9	0.289
Stage (*n*)			< 0.001
Ⅰ and Ⅱ	157	7	
Ⅲ	123	11	
Ⅳ	210	47	
Not available	15	1	
Tumor grade (*n*)			0.09
1	34	4	
2	97	9	
3	167	27	
Not available	207	26	
Survival (mean in months)	21.9 (20.6-23.2)	9.6 (8.1-11.1)	< 0.01

### Prognostic determinants

Parameters analyzed included age, gender, leukocytosis, thrombocytosis, tumor histology, tumor stage, mutation status and chemotherapy. The results of the univariate and multivariate analysis are illustrated below (Table 3). Gender [0.74 (0.61-0.90); *P*=0.03], p-Leukocytosis [2.38 (1.74-3.26); *P* < 0.001], p-Thrombocytosis [2.28 (1.5-3.46); *P* < 0.001], non-small cell histology [1.6 (1.30-2.03); *P* < 0.001], stage [4.4 (3.4-5.69); *P* < 0.001] were statistically related to patient's OS on univariate analysis. Multivariate analysis showed that gender [0.74 (0.60-0.91); *P*=0.01], stage [6.54 (4.91-8.70); *P* < 0.001], p-Leukocytosis [1.84 (1.32-2.57); *P* < 0.001], p-Thrombocytosis [1.64 (1.05-2.55); *P*=0.03] were independent prognostic determinants of OS in NSCLC.

**3:** Univariate and multivariate analysis of clinic-pathological variables with overall survival

Variables	Univariate			Multivariate	
	HR (95%CI)	*P* value		HR (95%CI)	*P* value
Age	1.01 (1.00-1.019)	0.05			
Gender	0.74 (0.61-0.90)	0.03		0.74 (0.60-0.91)	0.01
*EGFR* mutation	1.27 (0.53-3.02)	0.59			
Thrombocytosis	2.28 (1.5-3.46)	< 0.001		1.64 (1.05-2.55)	0.03
Leukocytosis	2.38 (1.74-3.26)	< 0.001		1.84 (1.32-2.57)	< 0.001
Adenocarcinoma	0.89 (0.74-1.08)	< 0.26			
Squamous cell	0.97 (0.79-1.18)	0.74			
Non-small cell	1.6 (1.30-2.03)	< 0.001		1.16 (0.92-1.47)	0.20
Stage	4.4 (3.4-5.69)	< 0.001		6.54 (4.91-8.70)	< 0.001
Chemotherapy	0.83 (0.68-1.00)	0.04		0.41 (0.34-0.51)	< 0.001

### Study outcomes

Patients with p-Thrombocytosis [(5±1.3) months; *P* < 0.001], p-Leukocytosis [(3±0.5) months; *P* < 0.001] and combined p-Leukothrombocytosis [(2±1.6) months; *P*=0.24] had significantly decreased median survival times compared to patients without p-Leuko/Thrombocytosis [(16±1.3) months](Figure 1). There was no statistical difference in survival of patients with combined Leukothrombocytosis compared to with those with either leukocytosis or thrombocytosis (*P*=0.79; 0.84 respectively). Median OS times of stage Ⅰ/Ⅱ NSCLC patients with p-Leuko/Thrombocytosis [(87±10.6) months] were not statistically different from those without p-Leuko/Thrombocytosis [(61±5) months](*P*=0.09). We did not have sufficient data on these patients' disease status to assess for disease-free survival (DFS).

**1:**
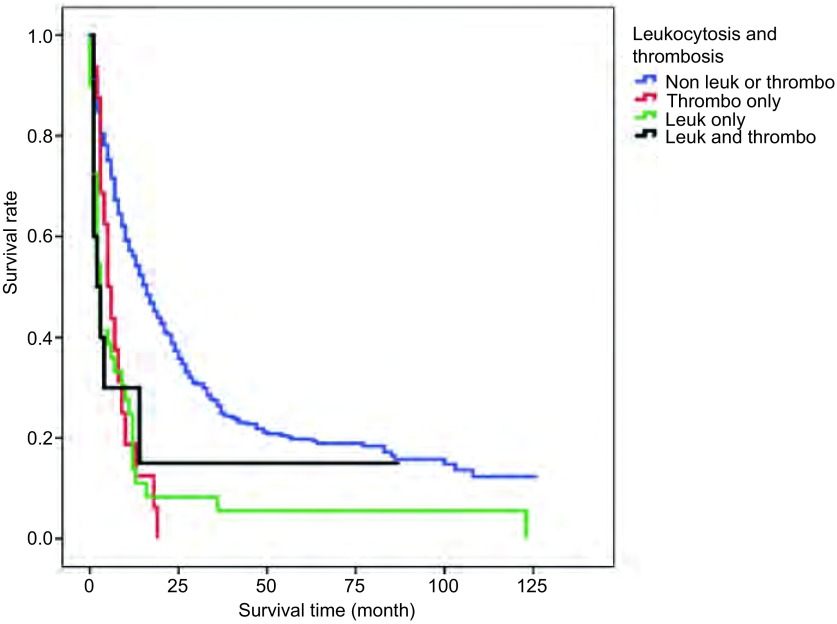
*Kaplan*-*Meier* survival curves comparing overall survival of various study groups

### Molecular marker status

NSCLC patients who had positive *EGFR* mutations were not more likely to present with Leuko/thrombocytosis compared to those with who did not have *EGFR* mutations (1/5 *vs* 8/55 respectively). Presence of *EGFR* mutation as an individual risk factor for leuko/thrombocytosis were not assessed due to small sample size. Survival comparisons between *EGFR* mutation positive patients with and without p-Leuko/Thrombocytosis could not be examined statistically due to small sample estimates. Other mutational types such as *ALK* mutation were too few in number to allow for any interpretation or analysis.

## Discussion

Based on our study, patients with either leukocytosis or thrombocytosis had a significantly decreased survival compared to patients with normal baseline blood counts at the time of diagnosis. Results of our study are in general agreement with the previously described studies which linked thrombocytosis and leukocytosis individually to poor prognosis in both resectable and non-resectable lung carcinoma^[4, 6, 10-14]^. These studies either did not identify their hematological parameter of interest as tumor related or only involved analysis of advanced stage lung cancer patients. Our study included all stages of NSCLC and involved analysis of patients with elevated blood counts considered to be paraneoplastic in origin (based on chart review).

We did not observe any statistical difference in survival of patients with combined p-Leukothrombocytosis syndrome when compared to those with either p-Leukocytosis or p-Thrombocytosis. We postulate that cumulative secretion of various tumor cytokine factors do not necessarily have an additive impact in worsening survival outcomes. Also, patients with p-Leukocytosis below 30, 000/mm^3^ had survival rates similar to those with levels above 30, 000/mm^3^. Survival outcomes may not be dictated by the degree of cell count elevation but by the presence or absence of hematological paraneoplasia.

More than 80% of patients with p-Leuko/Thrombocytosis, in our study, had stage Ⅲ or Ⅳ. It is currently unclear whether secretion of cytokine factors are primarily passive epiphenomena of inherent biological tumor aggression and or if they play an active role in contributing to tumor progression. However, local cytokines control the surrounding inflammatory milieu and have been demonstrated to have tumor and metastasis promoting effects in various clinical and basic models^[15]^.

Only 16% of our sample population with p-Leuko/Thrombocytosis were stage Ⅰ or Ⅱ. These patients did not have a statistically different overall survival compared to stage Ⅰ/Ⅱ patients without either paraneoplastic syndrome. A previous study demonstrated a prognostic impact of Thrombocytosis in stage Ⅰ/Ⅱ patients, which we could not replicate in our study^[14]^. Of note, the investigators noted that 11 of their 14 study patients with pre-operative thrombocytosis continued to have stable thrombocytosis post-surgery with a trend towards association between post-operative platelet count normalization and improved survival^[14]^. We were not able to gather post-operative blood count data on our study patients to assess for normalization. The effect of adequacy of surgical resection on the paraneoplastic syndrome and its association with survival outcomes is a matter of interest that warrants further study. IL-6 has a potent megakaryocytic effect and has shown to be responsible for thrombocytosis in CSF secreting tumors^[16]^.

Secretion of humoral factors has been associated more commonly with squamous cell histology type^[17, 18]^. Of the 50 patients with paraneoplastic leukocytosis, 6 had evidence of necrosis on pathological analysis. Two of these patients had squamous cell carcinoma, and the remaining 4 patients' tumor histopathology was classified under 'non-small cell carcinoma-nonspecific type'. Necrosis is characteristically seen in squamous cell histology^[19]^. All paraneoplastic leukocytosis patients with necrosis on gross or histopathology had counts greater than 24, 000/mm^3^. TNF-alpha produced by squamous cell carcinomas has been implicated to have a pathogenic role in the paraneoplastic effects of leukocytosis^[19]^. Cytokine driven leukocytosis and necrosis independently reflect aggressive neoplastic phenotype in SCC^[17, 18]^. Leukocytosis in these patients is less well explained by tissue necrosis as by cytokine secretion.

EGFR driven NSCLC tumors, in particular, have been demonstrated to secrete increased local concentrations of tumor promoting cytokines including IL-6, GCSF, and GM-CSF among others in the immediate tumor microenvironment^[20, 21]^. In our study, only one of nine patients with p-Leuko/Thrombocytosis had an *EGFR* mutation positive status. It remains a matter of speculation if increased tissue concentrations reach sufficient systemic levels to induce peripheral blood paraneoplasia. This data must be interpreted with caution since molecular marker status was available on 60 study patients only, given the fact that analysis for the above mutations/rearrangements is traditionally performed only in advanced stage adenocarcinoma which forms a small subset of our study population. The potential association warrants observation in larger sample sets. Similarly, survival comparisons could not be made between *EGFR* mutation positive patients with and without leuko/thrombocytosis due to small sample size.

Strengths of our study are that we analyzed specifically the relation between p-Leukocytosis/Thrombocytosis and survival. We conducted a chart review to carefully exclude all potential causes before labelling the hematological phenomena as paraneoplastic. Also, our study included all stages of NSCLC which helped identify the incidence and prognosis of the paraneoplasia even in earlier stages of NSCLC. The limitations of this study include the small sample size and retrospective study design. Due to limitations in sample size, we were not able to study the relation of molecular marker status and its impact on prognosis in NSCLC patients with p-Leuko/thrombocytosis. Another unavoidable limitation due to the retrospective nature of this study was that we ascribed the Leukothrombocytosis to paraneoplastic effect based on chart review. Since our study was conducted retrospectively, we could not support our conclusions with experimental data. However, our conclusions are well supported by data from previous experimental studies which demonstrated a very poor prognosis in NSCLC patients who had elevated tumor-related cytokine levels driving their Leukocytosis^[4]^.

## Conclusion

In conclusion, paraneoplastic Leukocytosis and paraneoplastic Thrombocytosis appear to have a prognostic significance in NSCLC. Our study demonstrated that the prognostic ability is confined to unresectable and advanced stage Ⅲ/Ⅳ NSCLC patients, but the small sample size in stage Ⅰ/Ⅱ patients indicates the need for further evaluation in a larger sample size. Blood counts are easily available and inexpensive lab markers that can serve as very useful clinical risk-stratifiers. The role of various cytokines in tumor pathobiology provides a rationale for identifying relevant cytokine factors responsible for the paraneoplasia and evaluating anti-cytokine therapies alongside traditional chemotherapy in an attempt to improve survival outcomes.

## Acknowledgements

All the authors had a role in writing this manuscript. Dr. Prajwal Boddu and Dr. Mebea Aklilu had full access to data in the study and contributed substantially to data interpretation and in writing the manuscript. Dana Villines was involved in study design, analysis and takes responsibility for the accuracy and integrity of the data analysis.

## Conflict of Interests

The authors whose names are listed immediately below certify that they have NO affiliations with or involvement in any organization or entity with any financial interest or non-financial interest in the subject matter or materials discussed in this manuscript. All the authors have a role in writing this manuscript.
